# Long noncoding RNAs predict the survival of patients with colorectal cancer as revealed by constructing an endogenous RNA network using bioinformation analysis

**DOI:** 10.1002/cam4.1813

**Published:** 2019-02-04

**Authors:** Hui Zhang, Zhuo Wang, Jianzhong Wu, Rong Ma, Jifeng Feng

**Affiliations:** ^1^ The Affiliated Cancer Hospital of Nanjing Medical University, Jiangsu Cancer Hospital Nanjing Jiangsu China; ^2^ Research Center for Clinical Oncology Jiangsu Cancer Hospital Nanjing Jiangsu China; ^3^ Jiangsu Institute of Cancer Research Nanjing Jiangsu China

**Keywords:** ceRNA, colorectal cancer, lncRNA, prognostic biomarker

## Abstract

Long noncoding RNAs (lncRNAs) are aberrantly expressed in various cancers types and can function as competing endogenous RNAs (ceRNAs), which promote and maintain tumor initiation and progression. In this study, we explored the functional roles and regulatory mechanisms of lncRNAs as ceRNAs in colorectal cancer and their clinical potential as biomarkers. The RNA sequencing profiles of patients with colorectal cancer were downloaded from TCGA database, and 62 lncRNAs, 30miRNAs, and 59 mRNAs were identified to comprise the ceRNA network (fold change > 2, *P* < 0.01). Functional enrichment analysis suggested that the target genes of the ceRNA network may be involved in the pathways related to cancer, including the signaling pathway that regulates the pluripotency of stem cells, wnt signaling pathway, hippo signaling pathway, basal cell carcinoma, and colorectal cancer. Univariate and multivariate Cox's proportional hazard regression model revealed that five (H19, MIR31HG, HOTAIR, WT1‐AS, and LINC00488) out of 62 lncRNAs were closely related to the overall survival (OS) (*P* < 0.05). Furthermore, the five‐lncRNA model could be an independent prognostic model in colorectal cancer. We computed for the risk function and constructed a risk score based on the five lncRNAs. Results showed that patients with high‐risk scores have poor survival rates. Additionally, combing the risk score and other clinicopathological features, we can better predict the patient's survival probabilities. Furthermore, we validate our model in the GSE38832 dataset. Collectively, our study has provided a deeper understanding of the lncRNA‐related ceRNA regulatory mechanism in CRC and identified five‐lncRNA model, which could be considered as candidate prognostic biomarkers and therapeutic targets.

## 1 INTRODUCTION

Colorectal cancer (CRC) is the third most commonly diagnosed malignancy and the fourth leading cause of cancer‐related mortality.^1^ Early diagnosis is crucial, and radical resection is the only curative therapy for cases where the cancer has not yet metastasized.^2^ Although the accumulation of molecules plays important roles in cancer processes, the heterogeneity of CRC renders difficulty in diagnosing and determining patient prognosis based on one factor.^3^ Thus, novel molecular networks that greatly optimize the use of therapies and benefit patients must be identified.

The multifaceted role of lncRNAs in CRC development has been extensively studied. LncRNAs participate in CRC development through the following ways: (a) as precursor of miRNAs or ceRNAs, (b) by interacting with proteins, (c) affecting gene transcription, and epigenetic mechanisms.^4^ The pathogenesis of lncRNAs in tumorigenesis and cancer development is further explained by the emergence of competing endogenous RNAs (ceRNAs) as an important class of posttranscriptional regulators that alter the expression of key tumorigenic or tumor suppressive genes through a microRNA‐mediated mechanism.^5^, ^6^, ^7^ Hence, the ceRNA network may serve as a biomarker for the diagnosis, prognosis, and prediction of therapeutic responses in CRC. The lncRNAs acting as ceRNAs have diverse biological functions that deserve further exploration.^8^

In this study, we collected the RNA sequencing (RNA‐Seq) data of 647 colorectal tumors and 51 adjacent non‐tumor samples from the TCGA database. The lncRNA expression profiles were combined with the clinical features, and a lncRNA‐miRNA‐mRNA ceRNA network was constructed. We then identified five‐lncRNA model with the potential to predict survival based on the ceRNA network and used these lncRNAs as novel candidate biomarkers for CRC.

## 2 MATERIALS AND METHODS

### 2.1 Patient information and data processing

The RNA‐Seq data and clinical information of CRC patients were completely downloaded from The Cancer Genome Atlas (TCGA) database (https://cancergenome.nih.gov/). The exclusion criteria were as follows: (a) without clinical or prognostic information, and (b) other malignancies in addition to CRC. Finally, 698 CRC samples including 647 tumor tissues and 51 matched normal tissues were enrolled for comprehensive integrated analysis. The data processing met the TCGA publication guidelines (https://cancergenome.nih.gov/publications/publicationguidelines).

### 2.2 Screening of differentially expressed RNAs (DERNAs)

The DERNA (DElncRNAs, DEmRNAs, and DEmiRNAs) data between tumor and normal samples were analyzed using “edgeR” package in R. Expression differences were characterized as fold change (FC) and associated *P*‐values. A log2|FC| > 2.0 and *P* < 0.01 were considered significant. The DERNA profiles were normalized by log2 transformation.

To further enhance the bioinformatics analysis reliability and facilitate subsequent verification, we screened the overlapping lncRNAs between the DElncRNA and GSE38832 RNA‐Seq data in the Gene Expression Omnibus (GEO) Datasets of NCBI dataset using Venn diagram for further study.

### 2.3 Constructing the lncRNA‐miRNA‐mRNA ceRNA network

To construct the ceRNA network, we first retrieved the human miRNA‐lncRNA interactions from the miRcode database (https://www.mircode.org/) and selected matched DElncNA and DEmiRNA. The target genes of matched DEmiRNAs were predicted using miRDB (https://www.mirdb.org/miRDB/), miRarBase, (https://mirtarbase.mbc.nctu.edu.tw/php/index.php) and TargetScan (https://www.targetscan.org/) online analysis tools. Only the overlapping target genes were identified to enhance the reliability of the bioinformatics analysis. Cytoscape v3.6.0 was used to construct and visualize the co‐expression network.^9^

### 2.4 Functional enrichment analysis

To further elucidate the biological function of ceRNA co‐expression, we performed Gene Ontology (GO) biological enrichment analysis through DAVID bioinformatics database and Kyoto Encyclopedia of Genes and Genomes (KEGG) pathways through the KOBAS database (https://kobas.cbi.pku.edu.cn). A significance level of *P* < 0.05 was set as the cutoff criteria.

### 2.5 Building a predictive model for prognosis and survival

The DElncRNAs were evaluated using univariate Cox's proportional hazard regression model in R to identify the prognostic signature. Only those with *P* value <0.05 were considered as candidate variables and entered into a stepwise multivariate Cox regression analysis. Then we construct a prognostic predictive model and obtained a combined prognosis score system (risk score) based on those DElncRNAs. The risk score was calculated as follows: Risk score = exp_lncRNA1 _× *β*_lncRNA1_ + exp_lncRNA2_ × *β*_lncRNA2_ + … exp_lncRNAn _× *β*_lncRNAn_), where, exp is the expression level; and *β* is the regression coefficient derived from the multivariate Cox regression model.^10^ The patients with CRC were categorized into high‐ and low‐risk groups according to its median.^11^ The differences in patients’ overall survival (OS) and disease‐free survival (DFS) between the two groups were evaluated by Kaplan‐Meier survival curve and log‐rank test analysis. The receiver operating characteristic (ROC) curve was used to assess the sensitivity and specificity of the lncRNA signatures in predicting survival.

Furthermore, using risk score and clinical information as covariates, univariate and multivariable Cox proportional hazards models were fitted to infer whether the risk score is an independent predictive factor. Hazard ratio (HR) and 95% confidence interval (CI) were also assessed.

### 2.6 Survival prognosis validation of the prognostic lncRNA model in GEO dataset

To verify the predictive performance of the prognostic predictive model, we further validated it in the GSE38832 dataset. Using the same model derived above, we formed high‐ and low‐risk group, respectively in the GSE38832. Kaplan‐Meier survival curve was drawn, and the ROC curve analysis was performed to evaluate the predictive power of the predictive model.

### 2.7 Combining risk score with clinical significance prognostic prediction for CRC

We then evaluated the prognostic value of different clinical features including American Joint Committee on Cancer (AJCC) TNM stage, invasive degree (T stage), lymph node status (N stage), metastasis (M stage). Moreover, we investigated the potential prediction ability of prognosis in CRC by combining risk score and clinical characteristics (low/high‐risk score + high/low stage) using a Kaplan‐Meier estimator and log‐rank test.

### 2.8 Statistical analysis

The relationship between the prognostic lncRNAs and clinical features was examined using Pearson correlation analysis. Difference of lncRNA expression between two groups was compared by Independent samples *t* test. All the hypothesis testing is two‐sided, and *P *value <0.05 was considered as statistically significant. Other analysis in this article was conducted in R version 3.4.2 (https://www.r-project.org/) with the following packages: “edgeR,” “pheatmap,” “forestplot,” “rms,” “ggplot2,” “survivalROC,” “survival.”

## 3 RESULTS

### 3.1 Identification of DERNAs in CRC

**Figure 1:**
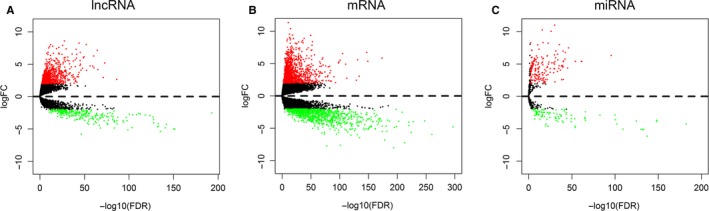
Volcano plot of differentially expressed RNAs in CRC patients. A, DElncRNAs; B, DEmRNAs; C, DEmiRNAs. The red dot represents up‐regulated RNA, and green dot represents down‐regulated RNA. log2|FC| > 2.0 and *P* < 0.01 as the selection criteria

A total of 698 samples, including 647 CRC tumor tissue samples and 51 adjacent non‐tumor tissue samples, were collected for this study. A total of 1143 DElncRNAs, including 888 up‐regulated and 255 down‐regulated lncRNAs, were identified in the CRC tissues and matched normal tissues according to the cutoff criteria (*P* < 0.01 and |log2FC| > 2.0). The volcano plot is presented in Figure 1A. We further obtained 276 DEmiRNAs (180 up‐regulated and 96 down‐regulated miRNAs) and 2151 mRNAs (1204 up‐regulated and 947 down‐regulated RNAs) from the TCGA database, and the results are shown in Figure 1B,C.

**Figure 2:**
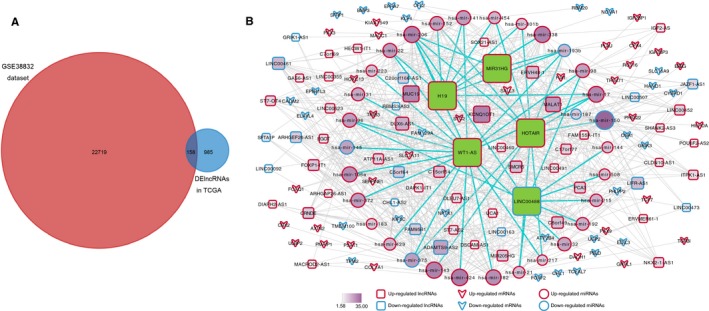
The ceRNAs network in CRC. A, The overlapping lncRNAs between GSE38832 dataset and DElncRNA in TCGA database; B, The lncRNA–miRNA–mRNA ceRNA network. Green diamonds, represented the five prognostic lncRNAs model. The size and color of a node represent the intensity of interaction in the network

Furthermore, by combining 1143 DElncRNAs derived from TCGA with GSE38832 dataset, we get 158 overlapping DElncRNAs for the subsequent study (Figure 2A).

### 3.2 lncRNA‐miRNA‐mRNA ceRNA network

To construct the ceRNA network, we assessed the target relationship between miRNAs and lncRNAs by using the miRcode among the aberrantly expressed lncRNAs and miRNAs. Furthermore, we predicted the target mRNAs of miRNAs through miRcode, miRDB, and miRarBase, and the overlapping genes were selected. The result showed co‐expression from 30 out of 276 miRNAs, 62 out of 158 lncRNAs, and 59 out of 2151 mRNAs (Figure 2B). The visualization of co‐expression was built using Cytoscape 3.6.0.

### 3.3 Function analysis

**Figure 3:**
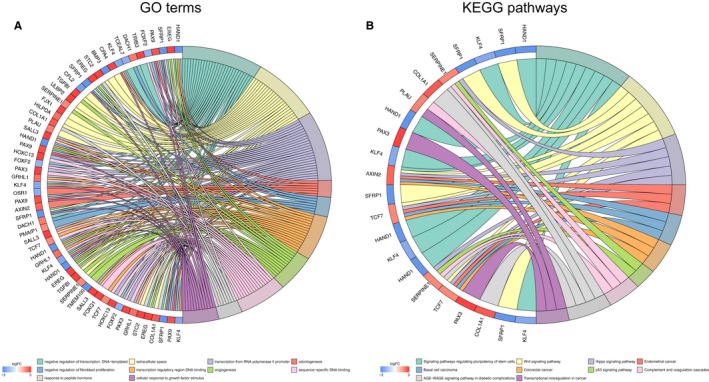
Enrichment analysis for differentially expressed mRNAs in ceRNAs network. A, The top 10 significant enriched GO analysis of mRNAs; B, The significant enriched KEGG pathways processes of mRNAs

To further elucidate the biological function of the ceRNAs, we performed the biological enrichment analysis through DAVID GO terms and KOBAS KEGG pathways related to the target gene. The KEGG pathways were significantly enriched in “signaling pathway regulating pluripotency of stem cells,” “wnt signaling pathway,” “hippo signaling pathway,” “basal cell carcinoma,” and “colorectal cancer”. The top five GO terms were “negative regulation of translation,” “extracellular space,” “transcription from RNA polymerase II promoter,” “odontogenesis,” and “negative regulation of fibroblast proliferation” (Figure 3).

### 3.4 Building a predictive model for prognosis and survival

**Figure 4:**
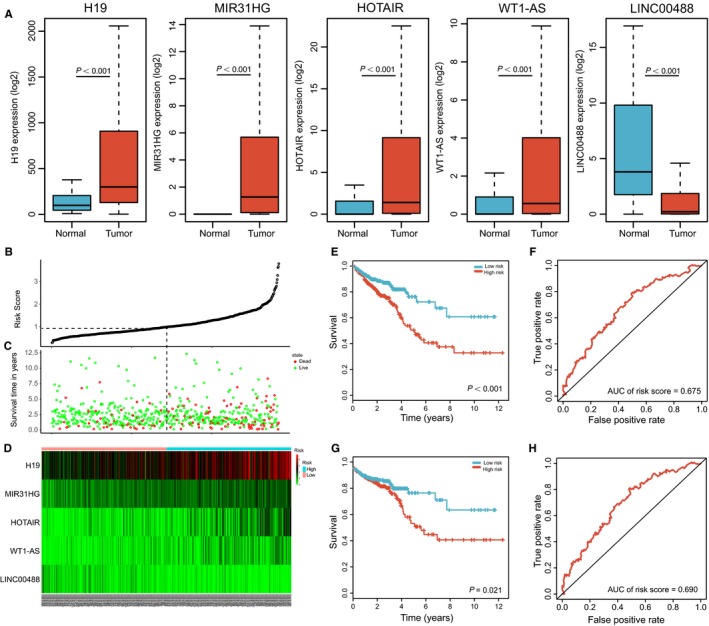
The five‐lncRNA model and its prognostic value for CRC patients. A, The expression level of the five lncRNAs in the tumor and normal tissues; B, The distribution of risk score of the model; C, Survival status and duration of cases; D, Heatmap of five lncRNAs expression profiles between low‐risk score and high‐risk score; E, G, Overall survival curves (E) and disease‐free survival (G) of CRC patients with low or high risk; F, H, The ROC for the risk score in predicting overall survival curves (F) and disease‐free survival (H)of CRC patients

We evaluated the association between lncRNAs expression and patients’ survival using univariate and multivariate Cox proportional hazard regression. Among the 62 DElncRNAs, five lncRNAs, namely, H19, MIR31HG, HOTAIR, WT1‐AS, and LINC00488 were screened out (Table 1, Figure 4A) and a predictive lncRNA model was constructed (Figure 4B‐D). The risk score which based on the five lncRNAs by their relative coefficient in multivariate Cox regression was calculated as: risk score = 0.0761 × exp_H19_ + 0.1690 × exp_MIR31HG_ + 0.0901 × exp_HOTAIR _+ 0.0976 × exp_WT1‐AS_ − 0.1551 × exp_LINC00488_. We then calculated the risk score for each patient and ranked them by increasing scores. Out of the 567 patients, 283 were classified in the high‐risk group and 284 in the low‐risk group based on the median score. Survival analysis was performed using the Kaplan‐Meier method with a log‐rank statistical test. The results showed that patients with high‐risk scores have significantly worse OS (*P* < 0.001, Figure 4E) and DFS than those with low‐risk scores (*P* = 0.021, Figure 4G). By calculating the area under the ROC curve (AUC) of risk score, we could predict the 5‐year survival of patients with colon adenocarcinoma (0.675 for OS and 0.690 for DFS) (Figure 4F,H).

**Table 1:** Overall information on the five prognostic lncRNAs

DElncRNAs	Ensembl ID	Univariate analysis		Multivariate analysis	
		HR	*P* value	HR	*P* value
H19	ENSG00000130600	1.098	0.008**	1.079	0.038*
MIR31HG	ENSG00000171889	0.821	0.033*	1.184	0.025*
HOTAIR	ENSG00000228630	1.111	0.005**	1.094	0.019*
WT1‐AS	ENSG00000183242	1.112	0.034*	1.103	0.041*
LINC00488	ENSG00000214381	0.754	0.048*	0.856	0.106

HR, hazard ratio.

* *P* < 0.05.

** *P* < 0.01.

*** *P* < 0.001.

### 3.5 Survival prognosis validation of the prognostic lncRNAs model in GEO dataset

**Figure 5:**
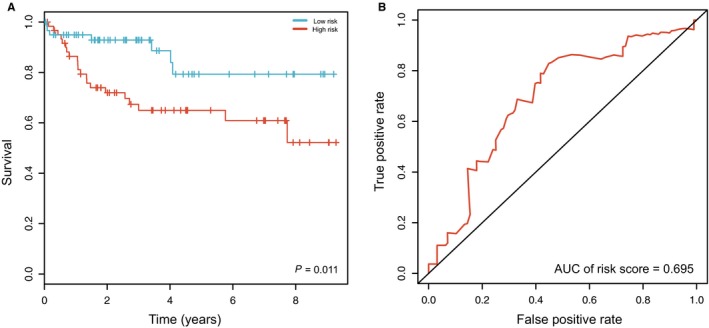
Validation of the prognostic lncRNAs model in GEO dataset. A, Kaplan‐Meier curve of the risk score for the OS of CRC patients in GSE38832 dataset; B, ROC curve of risk score for predicting 3‐y survival in GSE38832 dataset

To evaluate the survival predictive power of the five‐lncRNA model in CRC patients, this model was further tested in the GSE38832 dataset (n = 122). Using the same predictive model derived from the ceRNA network, 122 patients were classified into a high‐risk group (n = 61) and a low‐risk group (n = 61). As shown in Figure 5A, Patients in the low‐risk group had significantly longer overall survival time than those in the high‐risk group (*P* = 0.011). The AUC was 0.695 in the GSE38832 dataset (Figure 5B).

### 3.6 Combining risk score with clinical significance prognostic prediction for CRC

**Figure 6:**
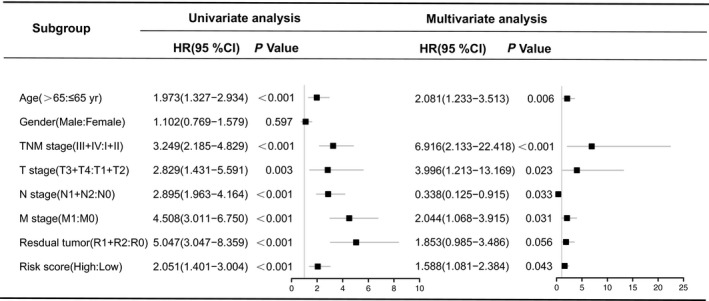
Univariate and multivariate Cox regression analysis in CRC patients. CI, confidence interval; HR, hazard ratio

The clinical correlation result showed that the risk score was significantly associated with TNM stage(0.002), T stage (*P* < 0.001), N stage (*P* = 0.004), T stage (*P* = 0.002) and Residual tumor (*P* = 0.030) (Table 2). We further correlated the clinical features with univariate and multivariate Cox regression analyses to test the effect of the five‐lncRNA model (high‐risk score vs. low‐risk score) on OS. In the univariate analysis, age (HR = 1.973, *P* < 0.001), TNM stage (HR = 3.249, *P* < 0.001), T stage (HR = 2.829, *P* = 0.003), N stage (HR = 2.895, *P* < 0.001), M stage (HR = 4.508, *P* < 0.001), residual tumor (HR = 5.047, *P* < 0.001), and risk score of the five‐lncRNA model (HR = 2.051, *P* < 0.001) were associated with the OS. In the multivariate analysis, the five‐lncRNA model (HR = 1.588, *P* = 0.043) could be an independent prognostic factor in patients with CRC (Figure 6).

**Table 2:** The correlations between the five‐lncRNA model and clinical features

Variable	Number	Risk score		
		Low risk	High risk	*P* value
Age				
≤65	251	132	119	0.293
＞65	316	151	165	
Gender				
Male	254	133	121	0.294
Female	313	150	163	
Tumor stage				
I ＋ II	300	168	132	0.002
III ＋ IV	249	106	143	
T stage				
T1 ＋ T2	114	76	38	<0.001*
T3 ＋ T4	452	207	245	
Lymph node status				
N0	317	176	141	0.004
N1‐2	247	107	140	
Metastasis				
M0	419	222	197	0.002
M1	80	27	53	
Residual tumor				
R0	408	218	190	0.030
R1/2	35	12	23	

* *P* < 0.05.

**Figure 7:**
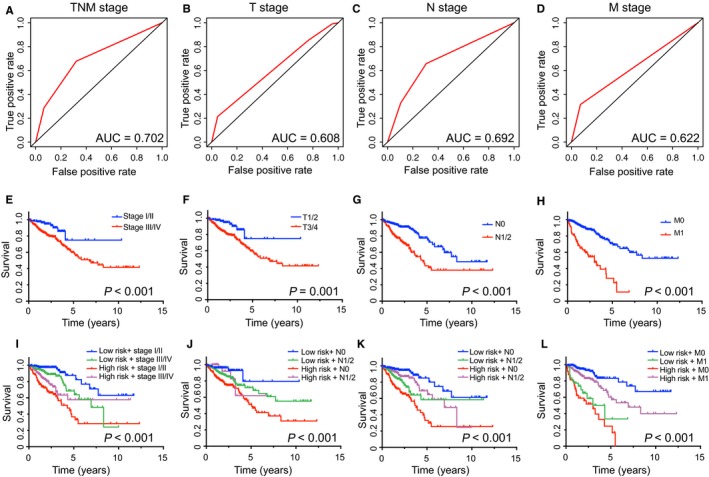
The prognostic value of different clinical features and risk score for OS of CRC patients

As expected, American Joint Committee on Cancer (AJCC) stage which was wildly applied in various tumors including CRC could predict the prognosis of patients effectively (Figure 7A‐H). Additionally, Kaplan‐Meier curves also showed that the patient's prognosis separated by risk score and TNM staging have significantly different (*P* < 0.001, Figure 7I‐L). Patients with lower risk score and tumor grade have obvious better prognosis.

**Figure 8:**
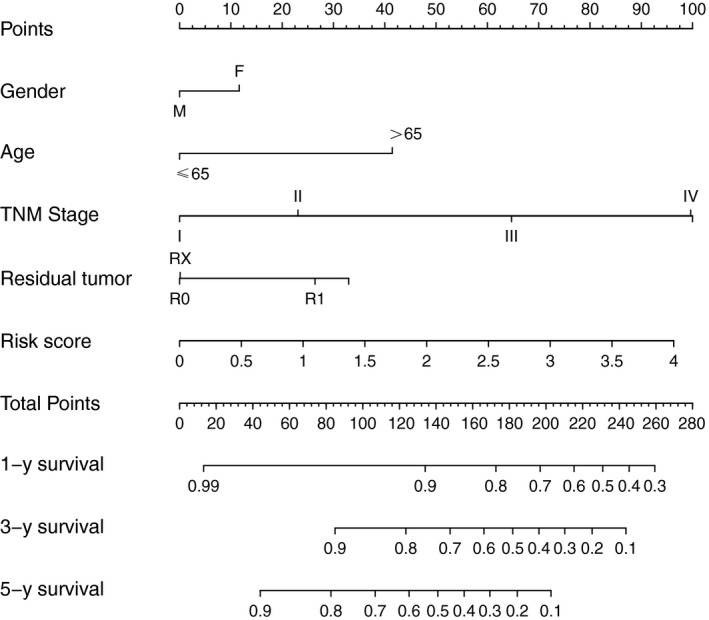
Nomograms to predict 1‐, 3‐ and 5‐y survival probability in CRC. Total points were calculated by adding up the corresponding points of each individual covariate on the points scale. Then 1,3‐ and 5‐y related survival probabilities were obtained by directly converting total points

**Figure 9:**
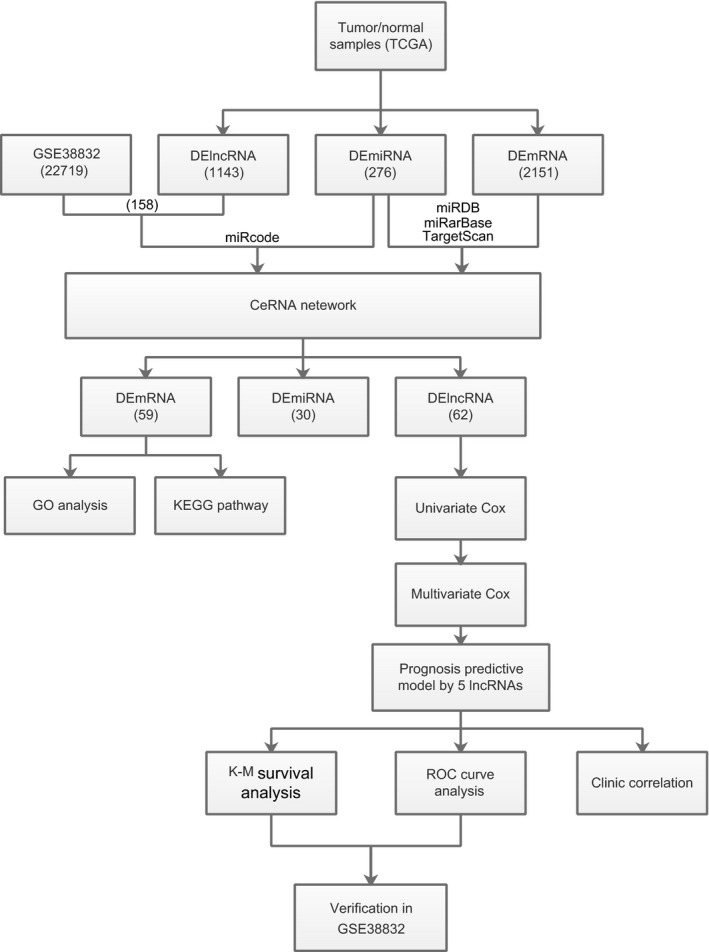
Flowchart of bioinformatics analysis

Therefore, we constructed a nomogram that integrated the risk score of five‐lncRNA model and clinicopathological features to predict survival probability of patients who had undergone surgical resection (Figure 8). Based on the risk score and clinicopathological features, we can better predict the patient's 1, 3, 5‐year survival probabilities (Figure 9).

## 4 DISCUSSION

Early diagnosis and radical resection are critical for CRC. The 5‐year survival rate is 90% when the localized disease is detected at an early stage. However, survival rates dramatically decrease to only 11% for patients with distant metastasis.^12^ CRC is a molecularly heterogeneous disease, and no single genetic “driver” is known to be superior in identifying aggressive disease.^13^ Thus, identifying novel molecular network biomarkers is needed to stratify patients for earlier detection and to improve targeted treatment options.

To date, the potential diagnostic and therapeutic targets of colorectal cancer research have focused primarily on the deregulation of protein‐coding genes. However, most biological characteristics, including tumorigenesis, arise from complex interactions of the cells with numerous constituents (eg, proteins, DNAs, RNAs, and small molecules) rather than with individual molecules.^14^ Thus, the regulatory networks of tumorigenesis must be clearly understood.

Accumulating evidence reveals that lncRNAs contain miRNA‐response elements and can compete with mRNAs for miRNAs. Hence, lncRNAs can act as ceRNAs and are implicated in multiple biological processes and tumorigenesis.^15^ Compared with protein‐coding genes, lncRNAs have significant advantages as diagnostic and prognostic biomarkers.^16^ Several studies have confirmed that the differentially expressed lncRNAs are closely related to the pathogenesis and prognosis of tumors and can be used as tumor‐associated predictors.^17^, ^18^

With the development of molecular techniques, new lncRNAs might prove to be vital components in the ceRNA network, which modulates other RNA transcripts.^19^ Tsang et al^20^ confirmed that the oncogenic functions of lncRNA H19 in CRC could be attributed to its ceRNA activity of sequestering miR‐675 and downregulating the expression of its target RB. FER1L4 could exert a tumor suppressive effect on colon cancer and partially acts as a ceRNA suppressing miR‐106a‐5p expression.^21^ LncRNA CCAT1 functions as a ceRNA participating in proliferation and apoptosis of human HCT‐116 and HCT‐8 cells.^22^ Zhou et al^23^ found that lincRNA‐ROR promotes the progression of colon cancer and holds prognostic value due to its association with miR‐145.

However, comprehensive analysis of large‐scale samples for calculating the prognostic value of the differentially expressed lncRNAs in patients with CRC has not yet been conducted. Here, we constructed a lncRNA‐miRNA‐mRNA ceRNA network in the TCGA database. We then identified five‐lncRNA (H19, MIR31HG, HOTAIR, WT1‐AS, and LINC00488) model in this network that was associated with the clinical outcome of CRC according to univariate and multivariate Cox proportional regression analyses. The aberrant expression of HOTAIR and H19 has been reported in various types of human cancer^24^, ^25^ and have been revealed as a negative prognostic factor for patients with colorectal cancer.^26^ H19 was reported to be the primary miRNA precursor of miR‐675 and could serve as the potential target for cancer therapy.^20^ HOTAIR also participates in gastric cancer as a ceRNA regulatory network.^27^ WT1‐AS is related to various cancers and may function as a tumor suppressor.^28^, ^29^ However, two novel lncRNAs (MIR31HG and LINC00488) have not been previously investigated and could be new prognostic indicators for patients with colon adenocarcinoma.

The AJCC TNM staging system is the preferred staging system for the management of CRC and could provide essential information for surgical solutions. By combining risk core and TNM staging, we could effectively predict the prognosis of patients, which further suggest that it may be responsible approach in predicting tumor occurrence and development.

Furthermore, the prediction model can be combined with other markers such as CEA to further improve the diagnostic efficiency of colon cancer. Additionally, based on the model and clinicopathological features, we can give patients a comprehensive score which better predicts the patient's 1, 3, 5‐year survival probabilities.

The results of the KEGG pathway involved in ceRNA network analysis showed that targeted genes were mainly enriched in “signaling pathway regulating pluripotency of stem cells,” “wnt signaling pathway,” “hippo signaling pathway,” “basal cell carcinoma,” and “colorectal cancer.” Self‐renewal and differentiation of stem cells are regulated by morphogenic pathways such as Wnt and Notch signaling.^30^ High Wnt pathway activity is important in determining the fate of cancer stem cells in CRC.^31^ Dysregulation of the Hippo pathway exerts a significant impact on cancer development including CRC.^32^

In summary, we successfully constructed a lncRNA‐associated ceRNA network in a large‐scale assembly of CRC samples and confirmed that the deregulation of the ceRNA network can lead to tumorigenesis. Furthermore, we constructed an independent survival prognostic model by analyzing the genome‐wide lncRNA expression profiles using a ceRNA network and discusses its clinical application value. The five‐lncRNA model could serve as potential prognostic indicator alone or in combination with other clinicopathological for patients with CRC. Compared with the previous literature,^33^, ^34^ we validate the prognostic model in the GEO database, which increases the reliability of the results. However, our study was limited by shortage of our clinical validation cohort. Besides, future functional investigations and molecular experiment are still required to explore the mechanisms underlying the roles of these lncRNAs in CRC.

## CONFLICT OF INTERESTS

No conflict of interest exists in the submission of this manuscript.

## References

[1] Torre LA , Bray F , Siegel RL , et al. Global cancer statistics, 2012. CA Cancer J Clin. 2015;65:87‐108. 25651787 10.3322/caac.21262

[2] Mármol I , Sánchez‐de‐Diego C , Dieste AP , Cerrada E , Yoldi M . Colorectal carcinoma: a general overview and future perspectives in colorectal cancer. Int J Mol Sci. 2017;18:1‐39. 10.3390/ijms18010197 PMC5297828 28106826

[3] Jacob H , Stanisavljevic L , Storli KE , Hestetun KE , OlavDahl , Myklebust et al. Identification of a sixteen‐microRNA signature as prognostic biomarker for stage II and III colon cancer. Oncotarget. 2017;8:87837‐87847. 29152124 10.18632/oncotarget.21237 PMC5675676

[4] Weng M , Di W , Yang C , et al. Non‐coding RNAs in the development, diagnosis and prognosis of colorectal cancer. Transl Res. 2016;181:108‐120. 27810413 10.1016/j.trsl.2016.10.001

[5] Salmena L , Poliseno L , Tay Y , Kats L , Pandolfi PP . A ceRNA hypothesis: the Rosetta Stone of a hidden RNA language? Cell. 2011;146:353‐358. 21802130 10.1016/j.cell.2011.07.014 PMC3235919

[6] Tang J , Zhuo H , Zhang X , et al. A novel biomarker Linc00974 interacting with KRT19 promotes proliferation and metastasis in hepatocellular carcinoma. Cell Death Dis. 2014;5:1‐13. 10.1038/cddis.2014.518 PMC4649834 25476897

[7] Zhou Xi , Gao Q , Wang J , Zhang X , Liu K , Duan Z . Linc‐RNA‐RoR acts as a “sponge” against mediation of the differentiation of endometrial cancer stem cells by microRNA‐145. Gynecol Oncol. 2014;133:333‐339. 24589415 10.1016/j.ygyno.2014.02.033

[8] Li F , Huang C , Li Q , Xianghua Wu . Construction and comprehensive analysis for dysregulated long non‐coding RNA (lncRNA)‐associated competing endogenous RNA (ceRNA) network in gastric cancer. Med Sci. 2018;24:37‐49. 10.12659/MSM.905410 PMC5761711 29295970

[9] Shannon P , Markiel A , Ozier O , et al. Cytoscape: a software environment for integrated models of biomolecular interaction networks. Genome Res. 2003;13:2498‐2504. 14597658 10.1101/gr.1239303 PMC403769

[10] Siegel R , Desantis C , Virgo K , et al. Cancer treatment and survivorship statistics, 2012. CA Cancer J Clin. 2013;62:220‐241. 10.3322/caac.21149 22700443

[11] Maher CA , White NM . The potential use of lncRNAs found in the 8q24 region as biomarkers for colon cancer. Ann Oncol. 2017;8:1688‐1698. 10.1093/annonc/mdx337 28838213

[12] Barabási A‐L , Oltvai ZN . Network biology: understanding the cell's functional organization. Nat Rev Genet. 2004;5:101‐113. 14735121 10.1038/nrg1272

[13] Wang Y , Chen Y , Hou J , et al. The emerging function and mechanism of ceRNAs in cancer. Trends Genet. 2016;32:211‐224. 26922301 10.1016/j.tig.2016.02.001 PMC4805481

[14] Hauptman N , Glavač D . Long non‐coding RNA in cancer. Int J Mol Sci. 2013;14:4655‐4669. 23443164 10.3390/ijms14034655 PMC3634483

[15] Augoff K , McCue B , Plow EF , Sossey‐Alaoui K . MiR‐31 and its host gene ln‐cRNA LOC554202 are regulated by promoter hypermethylation in triple‐negative breast cancer. Mol Cancer. 2012;11:1‐12. 22289355 10.1186/1476-4598-11-5 PMC3298503

[16] Li H , Beiqin Yu , Li J , et al. Overexpression of lncRNA H19 enhances carcinogenesis and metastasis of gastric cancer. Oncotarget. 2014;5:2318‐2329. 24810858 10.18632/oncotarget.1913 PMC4039165

[17] Zhou D , Sun T , Hacisuleyman E , et al. Integrative analyses reveal a long noncoding RNA‐mediated sponge regulatory network in prostate cancer. Nat Commun. 2016;15:1‐10. 10.1038/ncomms10982 PMC4796315 26975529

[18] Tay Y , Kats L , Salmena L , et al. Coding‐independent regulation of the tumor suppressor PTEN by competing endogenous mRNAs. Cell. 2011;147:344‐357. 22000013 10.1016/j.cell.2011.09.029 PMC3235920

[19] Karreth FA , Tay Y , Perna D , et al. In vivo identification of tumor‐suppressive PTEN ceRNAs in an oncogenic BRAF‐induced mouse model of melanoma. Cell. 2011;147:382‐395. 22000016 10.1016/j.cell.2011.09.032 PMC3236086

[20] Tsang WP , Ng EKO , Ng SSM , et al. Oncofetal H19‐derived mir‐675 regulates tumor suppressor RB in human colorectal cancer. Carcinogenesis. 2010;31:350‐358. 19926638 10.1093/carcin/bgp181

[21] Yue B , Sun Bo , Liu C , Zhao S , Dongyuan FY , Yan D . Long non‐coding RNA Fer‐1‐like protein 4 suppresses oncogenesis and exhibits prognostic value by associating with miR‐106a‐5p in colon cancer. Cancer Sci. 2015;10:1323‐1332. 10.1111/cas.12759 PMC4638023 26224446

[22] Li Bo , Shi C , Zhao J , Li B . Long noncoding RNA CCAT1 functions as a ceRNA to antagonize the effect of miR‐410 on the down‐regulation of ITPKB in human HCT‐116 and HCT‐8 cells. Oncotarget. 2017;8:92855‐92863. 29190961 10.18632/oncotarget.21612 PMC5696227

[23] Zhou P , Sun L , Liu D , Liu C , Sun L . Long non‐coding RNA lincRNA‐ROR promotes the progression of colon cancer and holds prognostic value by associating with miR‐145. Pathol Oncol Res. 2016;22:733‐740. 27071407 10.1007/s12253-016-0061-x

[24] Xin Yu , Li Z . Long non‐coding RNA HOTAIR: a novel oncogene. Mol Med Rep. 2015;12:5611‐5618. 26238267 10.3892/mmr.2015.4161

[25] Shi X , Sun M , Liu H , Yao Y , Song Y . Long non‐coding RNAs: a new frontier in the study of human diseases. Cancer Lett. 2013;339:159‐166. 23791884 10.1016/j.canlet.2013.06.013

[26] Kogo R , Shimamura T , Mimori K , et al. Long noncoding RNA HOTAIR regulates polycomb dependent chromatin modification and is associated with poor prognosis in colorectal cancers. Cancer Res. 2011;71:6320‐6326. 21862635 10.1158/0008-5472.CAN-11-1021

[27] Liu X‐H , Sun M , Nie F‐Q , et al. LncRNA HOTAIR functions as a competing endogenous RNA to regulate HER2 expression by sponging miR‐331‐3p in gastric cancer. Mol Cancer. 2014;13:92‐106. 24775712 10.1186/1476-4598-13-92 PMC4021402

[28] Lv L , Chen G , Zhou J , Li J , Gong J . WT1‐AS promotes cell apoptosis in hepatocellular carcinoma through down‐regulating of WT1. J Exp Clin Cancer Res. 2015;34:119‐128. 26462627 10.1186/s13046-015-0233-7 PMC4604772

[29] Pennucci V , Zini R , Norfo R , et al. Abnormal expression patterns of WT1‐as, MEG3 and ANRIL long non‐coding RNAs in CD34+ cells from patients with primary myelofibrosis and their clinical correlations. Leuk Lymphoma. 2015;56:492‐496. 24707949 10.3109/10428194.2014.910661

[30] Colak S , Medema JP . Cancer stem cells – important players in tumor therapy resistance. FEBS J. 2014;281:4779‐4791. 25158828 10.1111/febs.13023

[31] Vermeulen L , Felipe De Sousa E , Melo M , et al. Wnt activity defines colon cancer stem cells and is regulated by the microenvironment. Nat Cell Biol. 2010;12:468‐476. 20418870 10.1038/ncb2048

[32] Fa‐Xing Yu , Zhao B , Guan K‐L . Hippo pathway in organ size control, tissue homeostasis, and cancer. Cell. 2015;163:811‐828. 26544935 10.1016/j.cell.2015.10.044 PMC4638384

[33] Fan Q , Liu B . Discovery of a novel six‐long non‐coding RNA signature predicting survival of colorectal cancer patients. J Cell Biochem. 2018;119:3574‐3585. 29227531 10.1002/jcb.26548

[34] Xiong Y , Wang R , Peng L , et al. An integrated lncRNA, Zhang microRNA and mRNA signature to improve prognosis prediction of colorectal cancer. Oncotarget. 2017;8:85463‐85478. 29156733 10.18632/oncotarget.20013 PMC5689623

